# Uncinate fasciculus and its cortical terminals in aphasia after subcortical stroke: A multi-modal MRI study

**DOI:** 10.1016/j.nicl.2021.102597

**Published:** 2021-02-23

**Authors:** Binlong Zhang, Jingling Chang, Joel Park, Zhongjian Tan, Lu Tang, Tianli Lyu, Yi Han, Ruiwen Fan, Ying Gao, Jian Kong

**Affiliations:** a Department of Neurology, Dongzhimen Hospital, Beijing University of Chinese Medicine, Beijing, China; b Department of Psychiatry, Massachusetts General Hospital, Harvard Medical School, Boston, MA, USA; c Key Laboratory of Encephalopathy Treatment of Chinese Medicine, State Administration of Traditional Chinese Medicine of the Peoples Republic of China, Beijing, China; d Department of Radiology, Dongzhimen Hospital, Beijing University of Chinese Medicine, Beijing, China; e Department of Acupuncture and Moxibustion, Guang’anmen Hospital, China Academy of Chinese Medical Sciences, Beijing, China

**Keywords:** Aphasia, Subcortical stroke, Multimodal MRI, Uncinate fasciculus, Temporal pole, Disconnection theory

## Abstract

Aphasia, one of the most common cognitive impairments after stroke, is commonly considered to be a cortical deficit. However, many studies have reported cases of post subcortical stroke aphasia (PSSA). The pathology and recovery mechanism of PSSA remain unclear. This study aimed to investigate PSSA mechanism through a multimodal magnetic resonance imaging (MRI) approach and a two-session study design (baseline and one month after treatment). Thirty-six PSSA patients and twenty-four matched healthy controls (HC) were included. All patients had subcortical infarctions involving left subcortical white matter for 1 to 6 months. The patients underwent MRI scan and Western Aphasia Battery (WAB) examination before and after one month’s comprehensive treatment. Region-wise lesion-symptom mapping (RLSM), tractography, fractional anisotropy (FA), and amplitude of low-frequency fluctuations (ALFF) analysis were conducted. After MRI preprocessing and exclusion, FA analysis included 35 patients pre-treatment and 16 patients post-treatment. ALFF analysis included 30 patients pre-treatment and 14 patients post-treatment. We found: 1) the amount of damage in the left uncinate fasciculus (UF) was associated with WAB aphasia quotient (AQ); 2) the left UF FA and left temporal pole (TP) ALFF were decreased and positively correlated with WAB-AQ, spontaneous speech, and naming in PSSA patients; and 3) PSSA patients showed increased left TP ALFF when their language ability recovered after treatment. The left TP ALFF change was positively correlated with AQ change. Our results demonstrate the importance of left UF and left TP (one of the cortical terminals of the left UF) in PSSA pathology and recovery. These results may further provide support for the disconnection theory in the mechanism of PSSA.

## Highlights

• The left UF damage is associated with PSSA language impairment severity.
• The left UF FA is decreased and correlated with language impairment in PSSA.
• The left temporal pole ALFF is associated with PSSA language impairment and recovery.
• Our results support the disconnection theory in PSSA pathology and recovery.

## 1 Introduction

It is believed that the core brain regions associated with language are located at the left frontal and temporal cortical areas (Fedorenko and Thompson-Schill, 2014). However, a lot of studies have reported cases of aphasia after a single subcortical stroke (Choi et al., 2007, Radanovic and Mansur, 2017). The mechanism of these cases of aphasia remains unclear. Several theories have been proposed to explain the occurrence of post-subcortical-stroke aphasia (PSSA). Such theories include impairment in cortical circulatory dynamics after subcortical lesions (Radanovic and Mansur, 2017), disconnection of white matter connecting language-associated cortical regions (Stemmer and Whitaker, 2008), and the direct participation of subcortical grey matter in language processing (Kotz and Schwartze, 2010). However, the validity of these theories needs further investigation.

Multimodal magnetic resonance imaging (MRI) allows us to simultaneously investigate different aspects of brain structure and function non-invasively on patients to develop a comprehensive understanding of the underlying mechanism of PSSA (Bates et al., 2003, Jung et al., 2018, Park et al., 2013, Zhang et al., 2019). A three-dimensional structural T1-weighted image (3D-T1) can provide the precise location of a lesion region, and conducting lesion-symptom mapping (LSM) on 3D-T1 data is an effective way to reveal the relations between lesion areas and PSSA impairments (Bates et al., 2003). Additionally, applying diffusion tensor imaging (DTI) and resting-state functional MRI (RS-fMRI) allows for an in-depth investigation. Conducting tractography on DTI data enables the investigation of white-matter tract integrity (Park et al., 2013), and calculating RS-fMRI metrics may allow for the evaluation of gray matter neural process (Chen et al., 2018, Zhang et al., 2019). Particularly, amplitude of low-frequency fluctuations (ALFF), an index of low-frequency fluctuations, was used in this study due to its good test-retest reliability and replicability among commonly used RS-fMRI metrics (Chen et al., 2018). In addition, studies have linked ALFF with cerebral blood flow and task-evoked activation, and provided support for its physiological significance (Yuan et al., 2013, Zou et al., 2009).

Combining multimodal MRI technologies (LSM, tractography, and ALFF), this study aimed to investigated brain functional and structural changes associated with the pathology and recovery of PSSA using a two-session study design (baseline and one month after treatment). We hypothesized that PSSA would be associated with both functional and structural changes of the brain and that improvement in language ability would be correlated with changes in the key regions associated with PSSA.

## 2 Material and methods

### 2.1 Participants

Thirty-six PSSA patients and twenty-four healthy controls (HC) matched by age, gender, education, and handedness were included in this study. The patients enrolled were 35–80-year-old native Chinese speakers who were right-handed, as determined by the Edinburgh Handedness Inventory score ≥ 50 (Oldfield, 1971). All patients had pure subcortical infarctions for a duration of 1–6 months, as well as a diagnosis of aphasia based on a language test from the Western Aphasia Battery (WAB) (Kertesz, 1982). As in our previous study (Zhu et al., 2014), all patients received a score of ≥ 2 on the Boston Diagnostic Aphasia Examination (BDAE) severity rating scale (Goodglass, 2000), indicating that they could converse about familiar topics with help from listeners but had trouble when conveying their ideas. Patients were excluded from this study if they had other neurological, cognitive, or psychiatric disorders and/or if they were unable to enter the MRI scanner because of non-MRI compatible prostheses. The study was approved by the Institutional Review Board of Dongzhimen Hospital affiliated to Beijing University of Chinese Medicine, and all subjects signed informed consent forms.

All subjects underwent an MRI scan at baseline. Seventeen patients underwent a second MRI scan one month later. All seventeen patients received comprehensive treatment including language rehabilitation and acupuncture during this month. Demographic data and aphasia-related parameters for PSSA and HC subjects are presented in Table 1.

**Table 1:** Demographics and behavioral statistics of PSSA and HC subjects.

Characteristics	PSSA Baseline (n = 36)	PSSA who completed two sessions (n = 17)		HC (n = 24)
		Session 1	Session 2	
Age (years)	58.83 ± 9.76	54.59 ± 9.15		55.17 ± 6.13
Gender (male/female)	27/9	14/3		17/7
Education (years)	11.50 ± 3.19	12.24 ± 3.27		12.38 ± 2.45
Handedness (left/right)	0/36	0/17		0/24
Duration (days)	66.19 ± 35.19	68.24 ± 38.50		NA
Lesion location (left/bilateral)	24/12	11/6		NA
Lesion volume (mm^3^)	3064 ± 3619	3294 ± 4394		NA
WAB-AQ	69.31 ± 21.79	77.75 ± 11.96	88.68 ± 9.70**	NA
WAB-spontaneous speech	13.14 ± 4.31	14.32 ± 3.10	16.74 ± 2.73**	NA
WAB-auditory comprehension	148.83 ± 49.68	175.82 ± 19.82	191.12 ± 13.02**	NA
WAB-repetition	77.58 ± 26.78	85.35 ± 16.43	95.00 ± 4.89*	NA
WAB-naming	63.33 ± 26.82	72.35 ± 17.15	85.71 ± 15.40**	NA

Unless otherwise indicated, data are mean ± standard deviation. WAB: Western Aphasia Battery; AQ: aphasia quotient.

* P < 0.05 in a paired sample *t*-test; ** P < 0.01 in a paired sample *t*-test.

### 2.2 Behavioral evaluation

Before each MRI scan, the severity of language impairment of the patients was assessed by the WAB (Kertesz, 1982). This study focused on the aphasia quotient (AQ), representing the overall severity of language impairment of the patients, and four subtests of the WAB: spontaneous speech, auditory comprehension, repetition, and naming.

### 2.3 MRI data acquisition

All MRI data were collected on a 3T Siemens Verio whole-body scanner in Dongzhimen hospital. A 12-channel head coil with foam padding was used to restrict subjects’ head motion. For the RS-fMRI, the T2*-weighted functional images encompassing the whole brain was acquired with a gradient-echo echo-planar imaging (EPI) sequence (TR: 2000 ms, TE: 30 ms, FOV: 225 × 225 mm^2^, flip angle: 90°, matrix: 64 × 64 mm^2^, slice thickness: 3.5 mm, interslice gap: 0.7 mm, voxel size: 3.5 × 3.5 × 4.2 mm^3^, 31 interleaved axial slices, and 179 volumes). The 3D-T1 image was acquired using a multi-echo magnetization-prepared rapid gradient-echo (MPRAGE) sequence (TR: 1900 ms, TE: 2.13 ms, TI: 900 ms, slice thickness: 1 mm, flip angle: 9°, FOV: 256 × 256 mm^2^, voxel size: 1 × 1 × 1 mm^3^, and 176 sagittal slices). The DTI data were acquired with an axial single-shot spin echo diffusion-weighted EPI sequence (TR = 11000 ms, TE = 94 ms, FOV = 256 mm × 256 mm^2^, matrix = 128 × 128 mm^2^, b = 0 and 1000 s/mm^2^, slice thickness: 2 mm, voxel size: 2 × 2 × 2 mm^3^, 65 slices, and 30 gradient directions).

### 2.4 Lesion preprocessing

Lesions were manually drawn on the native T1 image space in MRIcroGL (https://www.mccauslandcenter.sc.edu/mricrogl/) by two neurologists (BZ and YH) who were blinded to the subjects’ WAB scores at the time of the lesion drawing. Then, the lesion maps were smoothed with a 3 mm full-width half maximum (FWHM) Gaussian kernel to remove jagged edges associated with manual drawing.

**Fig. 1:**
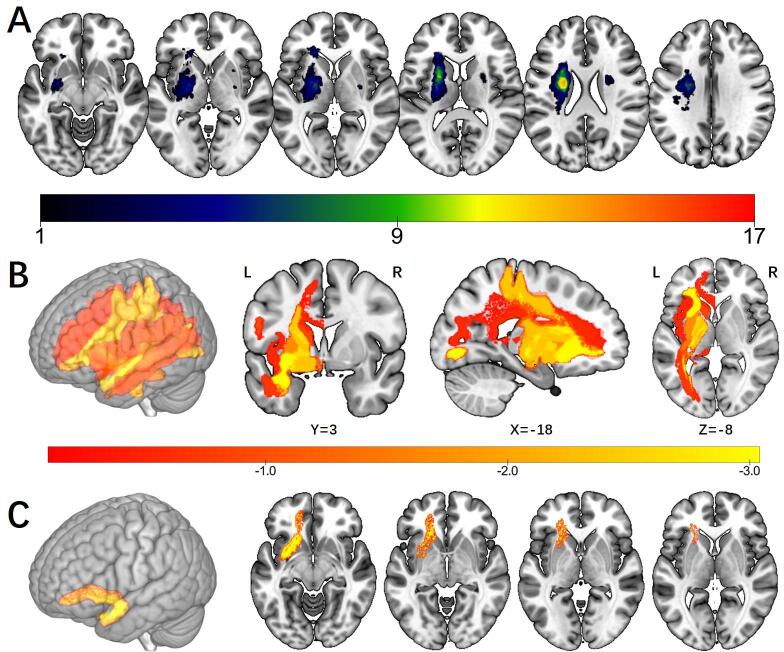
Overlap of lesions and results of region-wise lesion-symptom mapping analysis. A. Overlap of lesions in 36 baseline PSSA patients. The color indicates the number of participants having a lesion at a given location. B. Statistical maps of RLSM analysis. Nineteen regions were included in the RLSM analysis. The color indicates the z score of each region. C. Results of RLSM analysis. Only the left UF survived in the permutation test (Z = −3.04, permutation-corrected P < 0.05). Abbreviation: RLSM: region-wise lesion-symptom mapping; UF: uncinate fasciculus.

Spatial normalization of the lesion maps and T1 images were conducted using SPM 12 (https://www.fil.ion.ucl.ac.uk/spm/software/spm12/) and Clinical toolbox for SPM 12 (https://www.nitrc.org/projects/clinicaltbx/). For patients with a single left-side lesion, we performed enantiomorphic normalization as follows (Nachev et al., 2008): first, a mirrored image of the T1 image (reflected around the midline) was created. Then, a chimeric image based on the native T1 image was created with the lesioned tissue replaced by tissue from the mirrored image (the smoothed lesion map was used to modulate this blending). The chimeric image was warped to standard space using SPM12′s unified segmentation-normalization. The resulting spatial transform was applied to the actual T1 image as well as the lesion map. The normalized lesion map was then binarized using a 50% probability threshold (Nachev et al., 2008, Yourganov et al., 2018). For patients with bilateral lesions, we used cost function masking for the spatial normalization (Brett et al., 2001). The lesioned region was masked during the calculation of the normalization parameters in the cost function masking. Then, the lesion map was normalized by a continuation of the warping parameters derived from the unmasked brain areas. The overlap of lesions from 36 PSSA patients at baseline is presented in Fig. 1A.

### 2.5 Region-wise lesion-symptom mapping

To find out which damaged brain regions are responsible for language impairment in PSSA patients, we conducted a univariate region-wise lesion-symptom mapping (RLSM) analysis using NiiStat toolbox for Matlab (https://www.nitrc.org/projects/niistat/). The brain parcellation AALCAT (a combination of AAL and CAT (Catani and De Schotten, 2008) atlases) implemented in NiiStat was used to divide each normalized T1 image to 150 grey and white matter brain regions. Only regions where at least five participants had damage were included in the analysis. Similar to previous studies (Behroozmand et al., 2018, Fridriksson et al., 2018), a General Linear Model (pooled variance *t*-test, linear regression) with age, gender, education, and lesion volume as covariates were used to investigate the relationship between WAB AQ and the amount of damage in each brain region. A priori one-tailed hypothesis was made: brain damage leads to worse language performance. Statistical significance was determined by P < 0.05 (one-tailed) using permutation thresholding (5000 permutations) to control for multiple comparisons.

### 2.6 DTI processing

The DTI images were first visually inspected for apparent artifacts. Then, the images were preprocessed using FMRIB Software Library (FSL) version 6.0 (https://fsl.fmrib.ox.ac.uk/fsl/fslwiki/) and PANDA version 1.3.1 (http://www.nitrc.org/projects/panda/) with the following steps: skull removal using BET, correction of eddy current distortion, and calculation of the tensor matrix and fractional anisotropy (FA) using DTIFIT. Deterministic fiber tracking was applied using a fiber assignment by continuous tracking (FACT) algorithm with an angle threshold of 45° and an FA threshold of 0.2 ~ 1.

A tract-based spatial statistics (TBSS) was used for whole brain voxel-wise FA analysis (Smith et al., 2006). First, FA maps of all subjects were aligned to a standard space using an FMRIB58 template in MNI 152 standard space (https://fsl.fmrib.ox.ac.uk/fsl/fslwiki/FMRIB58_FA). Then, the aligned FA maps were averaged and thinned to produce a mean FA skeleton representing the center of all white matter tracts common to all subjects. The FA maps of all subjects were then projected to the mean skeleton (FA > 0.2) and fed into a general linear model for cross-subject statistics. Age, gender, and education were used as nuisance covariates. A nonparametric permutation testing (5000 permutations) with threshold-free cluster enhancement (TFCE) was utilized to threshold the results. Results were considered significant at P < 0.05 with TFCE corrected.

**Fig. 2:**
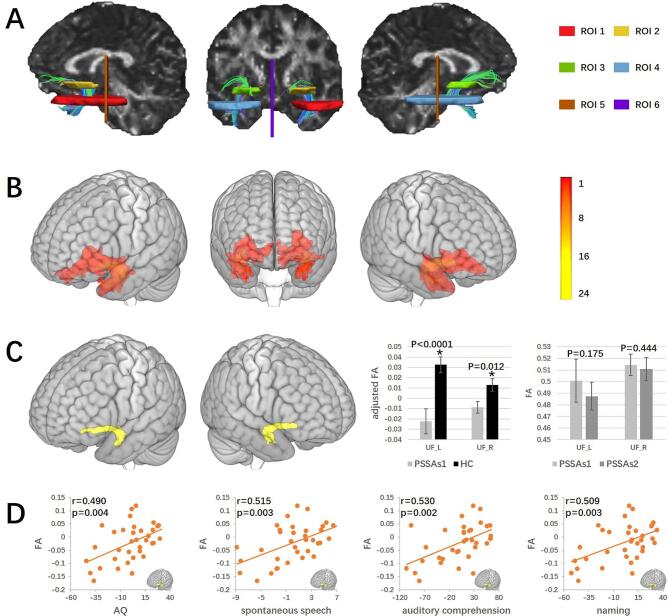
Diffusion tensor tractography results of the bilateral uncinate fasciculus. A. Regions of interest for reconstructing the bilateral UF and reconstructed bilateral UF from 1 healthy subject. ROIs 1 and 2 were used to include fibers of the left UF; ROIs 3 and 4 were used to include fibers of the right UF; ROIs 5 and 6 were used to remove unrelated fibers. B. Overlap of the bilateral UF for 24 HCs. The color indicates the number of participants having the UF passing through a given location. C. The templates of the bilateral UF with plots showing the comparison of bilateral UF FA values (mean ± standard deviation) between HCs and baseline PSSA patients, and between patients in session 1 and session 2. Baseline PSSA patients showed significantly reduced FA value in the bilateral UF as compared to HCs, but no significantly changed FA as compared to session 2. D. The correlations between left UF FA value and WAB scores in baseline PSSA patients. The left UF FA value was positively correlated with WAB AQ (p = 0.004), spontaneous speech (uncorrected p = 0.003, FDR p = 0.004), auditory comprehension (uncorrected p = 0.002, FDR p = 0.004), and naming (uncorrected p = 0.003, FDR p = 0.004) scores in PSSA patients. Note that due to partial correlations, axes show the residuals of parameters. Abbreviation: UF: uncinate fasciculus; FA: fractional anisotropy. ‘*’ indicates statistical significance (P < 0.05).

Based on the results of RLSM (Fig. 1C, showing that only damage in the left uncinate fasciculus (UF) predicted language impairment), we chose the bilateral UF as regions of interest (ROI) to further explore the relationship between UF integrity (using FA (Conturo et al., 1996) as the measurement) and language impairment. A two-ROI approach (Catani and De Schotten, 2008) was used to dissect each side of the UF including the anterior temporal lobe and external/extreme capsule (Fig. 2A). Two exclusion ROIs (Sobhani et al., 2015) were used to remove unrelated fibers from each side of the UF based on existing anatomic knowledge (Fig. 2A). A template-based automated quantification method was used to extract UF FA values of all subjects (Park et al., 2013). The UF ROIs were first manually delineated in each individual HC subject’s native space. Tractography was performed in all HCs. The FA maps and reconstructed tracts were coregistered into MNI space using a 2-step method: first, the FA map of each subject was coregistered to FMRIB58 template in MNI 152 standard space and resliced to 2*2*2 mm^3^. Non-linear normalization was conducted using FNIRT. Second, the affine parameter from the normalization was used to register the tracts to MNI space and resliced to 2*2*2 mm^3^. The resulting bilateral UF of all HCs were binarized and used to produce a group-level overlap map (Fig. 2B). The group-level overlap map was then binarized at a threshold of half of the number of HCs to serve as the bilateral UF template (Fig. 2C). The mean FA values were then extracted from the UF templates for all subjects and used to explore their correlations with language scores.

The statistical analysis of UF FA was performed in SPSS version 24.0. A General Linear Model approach was used to calculate the group FA difference between PSSA patients and HCs for each side of the UF, with age, gender, and education as covariates. A paired sample *t*-test was conducted to compare UF FA value differences between PSSA patients in session 1 and session 2.

### 2.7 Functional image processing

We applied amplitude of low-frequency fluctuation (ALFF) (Zhang et al., 2019) to explore brain functional changes in PSSA patients. Functional data preprocessing and statistical analysis were performed in DPABI version 3.1 (http://rfmri.org/dpabi) and SPM12. Preprocessing steps included the removal of the initial ten volumes; slice-timing correction; realignment; skull strip; normalization; nuisance regression using WM, CSF, linear trend, and head motion (Friston 24-parameter model); and spatial smoothing using 6 mm FWHM. Specifically, during normalization, the mean fMRI volume was aligned to the corresponding normalized T1-weighted image to compute the spatial transformation between the fMRI data and the lesion mask (Yourganov et al., 2018). After a fast Fourier transformation, ALFF was calculated as the mean of amplitudes within 0.01–0.10 Hz. The ALFF map was z-transformed (minus mean whole brain ALFF and then divided by standard deviation) prior to subsequent analysis (Zuo et al., 2010). The whole brain ALFF comparison was performed on a brain grey matter mask where all the lesioned voxels were excluded. The brain mask was created using the subtraction of the grey matter mask implemented in DPABI and the binarized lesion overlap map of the PSSA subjects. Subjects whose head motion evaluated by mean framewise displacement (FD) exceeded 0.2 mm were excluded from the analysis (Jenkinson et al., 2002).

We then conducted a two-sample *t*-test to compare whole-brain ALFF between PSSA patients (pre-treatment) and HCs (including age, gender, and education as covariates). Permutation testing with threshold-free cluster enhancement (TFCE) was utilized to threshold our results at p < 0.05 corrected. The TFCE was conducted in PALM package (Winkler et al., 2016) in DPABI with 5000 permutations and a cluster-forming threshold of z > 2.3. The ALFF value in the survived clusters was used to conduct correlation analysis. Then, the clusters related to language scores were used as ROIs to extract ALFF values in the paired *t*-test of PSSA patients in session 1 and session 2.

### 2.8 Partial correlation analysis

To explore the correlation between FA/ALFF values and language scores, partial correlation analysis was performed in SPSS with age, gender, education, and lesion volume as covariates. The WAB AQ was the primary outcome and the four WAB subtests were the secondary outcomes in the correlation analysis. All results were corrected for multiple comparisons using a false discovery rate (FDR) threshold of p < 0.05.

## 3 Results

### 3.1 Demographic information

Thirty-six PSSA patients and twenty-four HCs were included at baseline (Table 1). Seventeen PSSA patients completed the second scan. Nineteen patients did not have a second session due to unavailability. For the seventeen patients, their WAB AQ, spontaneous speech, auditory comprehension, repetition, and naming scores significantly increased in the second session.

### 3.2 Lesion analysis results

The lesions in the 36 PSSA patients were all subcortical (24 left-side and 12 bilateral) (Fig. 1A). After the exclusion of regions where less than five participants had damage, nineteen regions were included in the RLSM analysis (Fig. 1B). Only the left UF survived in the permutation test (z = −3.04, permutation-corrected P < 0.05), indicating that damage to the left UF predicts lower WAB AQ score (Fig. 1C).

### 3.3 DTI analysis results

One patient was excluded due to apparent artifacts. Thirty-five patients and twenty-four HCs were included in the comparison between PSSA and HC subjects. Sixteen patients were included in the comparison between PSSA patients before (PSSAs1) and after (PSSAs2) treatment. No significant result was found in the FA whole brain comparison between PSSA and HC, or between PSSA before and after treatment.

Based on the results of RLSM, we chose the bilateral UF as ROIs for further exploration. The FA values of the bilateral UF were significantly decreased in PSSA patients (Fig. 2C). We also found that the FA value of the left UF was positively correlated with WAB AQ, spontaneous speech, auditory comprehension, and naming scores in PSSA patients (Fig. 2D). However, we did not find a significant difference in bilateral UF FA values in PSSA patients before and after treatment (Fig. 2C).

### 3.4 ALFF analysis results

Two PSSA patients were excluded due to poor fMRI data quality (the scan did not cover the whole brain). Four patients were excluded due to excessive head motion. Thirty patients and twenty-four HCs were included in the ALFF comparison between PSSA and HC subjects, and fourteen patients were included in the ALFF comparison between PSSA patients before and after treatment (PSSAs1 vs PSSAs2). Head motion evaluated by mean FD was not significantly different (p = 0.695) between patients (0.095 ± 0.048, mean ± SD) and HCs (0.086 ± 0.043) and not significantly different (p = 0.429) between PSSAs1 (0.088 ± 0.044) and PSSAs2 (0.103 ± 0.048).

**Fig. 3:**
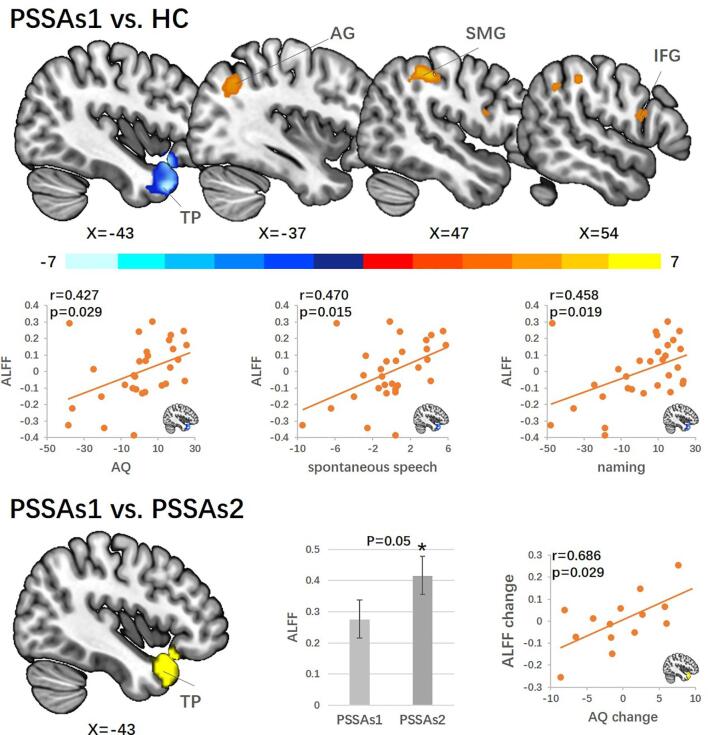
Results of amplitude of low-frequency fluctuations analysis. The comparison between baseline PSSA patients and HCs are shown at the top. Compared to HCs, PSSA patients showed decreased ALFF in the left TP and increased ALFF in the left AG, right SMG, and right IFG. Left TP ALFF value was positively correlated with WAB AQ (p = 0.029), spontaneous speech (uncorrected p = 0.015, FDR p = 0.038), and naming (uncorrected p = 0.019, FDR p = 0.038) scores. The comparison between PSSA patients in session 1 and session 2 was shown on the bottom. PSSA patients showed significantly increased ALFF in the left TP (p = 0.05) after one month. The left TP ALFF change is positively and significantly correlated with WAB AQ change (p = 0.029) in the patients. Note that due to partial correlations, axes show the residuals of parameters. Abbreviations: ALFF: amplitude of low-frequency fluctuations; TP: temporal pole; AG: angular gyrus; SMG: supramarginal gyrus; IFG: inferior frontal gyrus. ‘*’ indicates statistical significance.

We found significantly decreased ALFF in the left temporal pole (TP) and increased ALFF in the right supramarginal gyrus (SMG), right inferior frontal gyrus (IFG), and left angular gyrus (AG) in PSSA patients as compared to HCs. ALFF in the left TP was positively correlated with AQ, spontaneous speech, and naming scores in PSSA patients (Fig. 3 and Table 2). Then, we extracted ALFF values in PSSAs2 based on the binary mask of the left TP cluster survived in the PSSA and HC comparison. ALFF in the left TP was significantly increased in PSSAs2 as compared to PSSAs1 (p = 0.05). ALFF value change in the left TP was positively correlated with AQ score change in PSSA patients (Fig. 3 and Table 2). We also explored ALFF changes in the right SMG, right IFG, and left AG but found no significant change.

**Table 2:** Results of ALFF analysis.

Contrast	Regions	Peak MNI			Peak z-value	Voxels
		X	Y	Z		
PSSA > HC	SMG_R	48	−33	45	6.19	68
	AG_L	−36	−63	42	4.51	66
	IFG_R	54	9	18	4.04	43
PSSA < HC	TP_L	−42	18	−36	−6.68	433

Results reported for whole brain ALFF comparison between PSSA (before treatment, n = 30) and HC subjects with age, gender, and education as covariates. L: left; R: right; SMG: supramarginal gyrus; AG: angular gyrus; IFG: inferior frontal gyrus; TP: temporal pole.

## 4 Discussion

Using multimodal MRI approaches, we investigated the pathology and recovery mechanism of PSSA. We found that 1) WAB AQ score was predicted by the amount of damage in the left UF; 2) the FA in the bilateral UF was decreased in PSSA patients as compared to HCs. The left UF FA value was positively correlated with WAB AQ and other language subtest scores in PSSA patients; and 3) PSSA patients have decreased ALFF in the left TP (one of the cortical terminals of the left UF). The ALFF value in the left TP increased significantly after one month’s recovery, and the increase was positively correlated with AQ score change. Our results demonstrate the importance of left UF integrity and left TP low-frequency oscillation in PSSA pathology and recovery.

Current theories for the mechanism of PSSA include white matter disconnection, direct participation of subcortical grey matter, and impairment of cortical circulatory dynamics. The direct participation theory suggests that subcortical grey matter (e.g., thalamus and caudate) is directly involved in language processing (Kotz and Schwartze, 2010). Supporting this theory, studies have revealed activation of the caudate nucleus and putamen during word-generation tasks but not during nonsense syllable generation (Crosson et al., 2003). Nevertheless, most current language models do not contain any subcortical grey matter (Fedorenko and Thompson-Schill, 2014, Hagoort, 2013, Hickok and Poeppel, 2007), which calls for further investigation.

The cortical circulatory dynamics impairment theory suggests that PSSA language impairment is secondary to hypoperfusion in cortical territories of the injured artery (Radanovic and Mansur, 2017). This theory is supported by a previous study in which the authors found that all the PSSA patients within 24 h of stroke onset showed cortical hypoperfusion and that the resolution of aphasia is associated with restored cortical perfusion (Hillis et al., 2002). However, whether findings detected within 24 h of stroke onset can be extended to PSSA patients after a longer duration remains to be answered.

The disconnection theory suggests that disconnection among language associated cortical areas caused by subcortical white matter damage is the pathology of PSSA (Stemmer and Whitaker, 2008). This theory is supported by the increasing attention of the participation of subcortical white matter fibers in current language models (e.g. superior longitudinal fasciculus, arcuate fasciculus, and UF) (Friederici, 2015). For instance, the dual stream language model suggests two white matter pathways in language processing, a ventral pathway critical for auditory comprehension and a dorsal pathway important for speech production (Fridriksson et al., 2018). Our results, which emphasized the left UF and its cortical termination TP in PSSA pathology, provide direct support for the disconnection theory. Furthermore, the association between increased TP ALFF and increased AQ score in PSSA patients suggests that increased low-frequency oscillation of the disconnected cortex may act as an important marker reflecting the recovery of language impairment in PSSA.

We found that the UF plays an important role in the pathology of PSSA. This result is consistent with studies in which the authors found that the left UF may participate in both language production and comprehension processes. For instance, previous studies showed that apparent diffusion coefficient decrease in the left UF was correlated with aphasia severity (Zavanone et al., 2018), left UF integrity is positively correlated with naming and comprehension scores in aphasia patients (Harvey et al., 2013, Powers et al., 2013), left UF integrity is a predictor of speech fluency and semantic processing scores in post-stroke aphasia patients (Basilakos et al., 2014), and the UF is an important component in the ventral stream (Fridriksson et al., 2016).

We also found that ALFF value in the left TP was decreased and positively correlated with AQ, spontaneous speech, and naming scores in PSSA patients; the decreased left TP ALFF was significantly increased after one month’s recovery and the change of left TP ALFF was positively correlated with AQ score change in PSSA patients. Our results are consistent with a previous study suggesting that atrophy of the left TP is strongly associated with naming impairment in aphasia patients (Mesulam et al., 2013). Another study found that left TP involvement is correlated with impairments in speech fluency and naming in seizure-induced language disturbance (Trebuchon et al., 2018). Our results suggest that TP ALFF is not only involved in the PSSA pathology, but also sensitive to the development of language function, which supports using the region as an objective marker to monitor clinical improvement in patients with PSSA.

Our results suggest that the left UF and TP are involved in speech production, which may not be in line with classical language models. Traditionally, the left TP is considered to be a semantic hub and responsible for sentence-level composition (Ralph et al., 2010, Rogalsky, 2015), and the left UF is considered to be involved in the proper naming process and semantic processing (Papagno, 2011). However, an increasing number of studies have found an association between the left UF/TP and speech fluency impairment (Basilakos et al., 2014, Fridriksson et al., 2013, Trebuchon et al., 2018). Also, a recent study found that the left UF is a part of the extended connectome for Broca’s area, which provides structural support for the involvement of the left UF in language production (Lemaire et al., 2013). Another study found that the combinatory process in the left TP is an early process in language production (Pylkkänen et al., 2014). Thus, our results along with studies from other groups provide support for the involvement of the left UF and TP in the speech production process.

We also found increased ALFF in the left AG, right SMG, and right IFG in PSSA patients. The SMG and AG clusters in this study are both a part of the temporal-parietal junction (TPJ). The left TPJ is important for the auditory-to-articulation process in the dual stream model (Hickok and Poeppel, 2007). The right IFG cluster in this study is the homologous area of Broca’s area, which is a classical area responsible for speech production (Hage and Nieder, 2016). These results suggest that subcortical lesions can produce widespread cortical dysfunction, and increased ALFF in the bilateral cortical area may contribute to functional cortical reorganization in PSSA patients.

There are several limitations to this study. First, the sample size in the second session of PSSA patients (n = 17) is relatively small and the 17 patients shows a gender imbalance (14 male and 3 female). In addition, the small sample size prevents us from investigating the potential gender effect. Studies with a large sample size is needed to 1) further validate our finding and 2) explore the potential gender effect. Second, the patients who completed two sessions received a comprehensive treatment. Whether the increased TP low-frequency oscillation reflects a natural or treatment-induced recovery in PSSA needs further investigation.

## 5 Conclusions

In conclusion, our results demonstrate the importance of decreased left uncinate fasciculus integrity and left temporal pole (one of the cortical terminals of the left uncinate fasciculus) low-frequency oscillation in the pathology of PSSA, as well as the crucial role of increased left temporal pole low-frequency oscillation in the recovery of language impairment in PSSA. Our findings provide support for the disconnection theory in the pathology and recovery mechanism of PSSA.

## CRediT authorship contribution statement

**Binlong Zhang:** Conceptualization, Formal analysis, Investigation, Methodology, Project administration, Visualization, Writing - original draft, Writing - review & editing. **Jingling Chang:** Conceptualization, Funding acquisition, Investigation, Project administration, Supervision, Writing - review & editing. **Joel Park:** Writing - original draft, Writing - review & editing. **Zhongjian Tan:** Data curation, Writing - review & editing. **Lu Tang:** Investigation, Writing - review & editing. **Tianli Lyu:** Data curation, Writing - review & editing. **Yi Han:** Formal analysis, Writing - review & editing. **Ruiwen Fan:** Investigation, Writing - review & editing. **Ying Gao:** Conceptualization, Funding acquisition, Investigation, Project administration, Supervision, Writing - review & editing. **Jian Kong:** Conceptualization, Formal analysis, Methodology, Investigation, Supervision, Validation, Writing - review & editing.
